# Influence of the Rare Earth Cation on the Magnetic Properties of Layered 12R-Ba_4_M^4+^Mn_3_O_12_ (M = Ce, Pr) Perovskites

**DOI:** 10.1021/acs.chemmater.3c03014

**Published:** 2024-03-09

**Authors:** Michael J. Dzara, Arthur C. Campello, Aeryn T. Breidenbach, Nicholas A. Strange, James Eujin Park, Andrea Ambrosini, Eric N. Coker, David S. Ginley, Young S. Lee, Robert T. Bell, Rebecca W. Smaha

**Affiliations:** † National Renewable Energy Laboratory, Golden, Colorado 80401, United States; ‡ Stanford Institute for Materials and Energy Sciences, SLAC National Accelerator Laboratory, Menlo Park, California 94025, United States; § Department of Applied Physics, Stanford University, Stanford, California 94305, United States; ∥ Department of Physics, Stanford University, Stanford, California 94305, United States; ⊥ Stanford Synchrotron Radiation Lightsource, SLAC National Accelerator, Laboratory, Menlo Park, California 94025, United States; # Sandia National Laboratories, PO Box 5800, Albuquerque, New Mexico 87185, United States

## Abstract

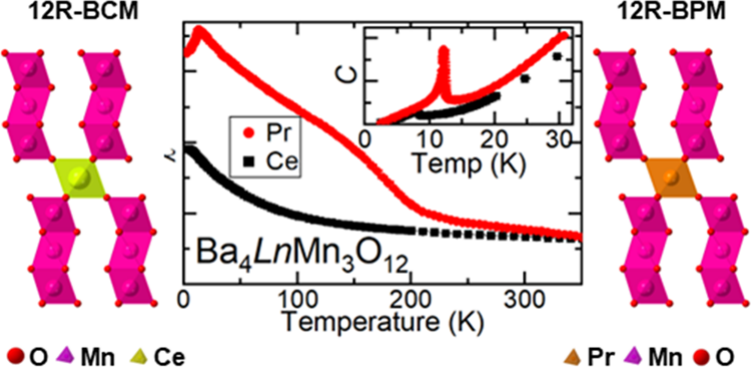

Material design is increasingly used to realize desired functional properties, and the perovskite structure family is one of the richest and most diverse: perovskites are employed in many applications due to their structural flexibility and compositional diversity. Hexagonal, layered perovskite structures with chains of face-sharing transition metal oxide octahedra have attracted great interest as quantum materials due to their magnetic and electronic properties. Ba_4_MMn_3_O_12_, a member of the “12R” class of hexagonal, layered perovskites, contains trimers of face-sharing MnO_6_ octahedra that are linked by a corner-sharing, bridging MO_6_ octahedron. Here, we investigate cluster magnetism in the Mn_3_O_12_ trimers and the role of this bridging octahedron on the magnetic properties of two isostructural 12R materials by systematically changing the M^4+^ cation from nonmagnetic Ce^4+^ (f^0^) to magnetic Pr^4+^ (f^1^). We synthesized 12R-Ba_4_MMn_3_O_12_ (M= Ce, Pr) with high phase purity and characterized their low-temperature crystal structures and magnetic properties. Using substantially higher purity samples than previously reported, we confirm the frustrated antiferromagnetic ground state of 12R-Ba_4_PrMn_3_O_12_ below *T*_N_ ≈ 7.75 K and explore the cluster magnetism of its Mn_3_O_12_ trimers. Despite being atomically isostructural with 12R-Ba_4_CeMn_3_O_12_, the f^1^ electron associated with Pr^4+^ causes much more complex magnetic properties in 12R-Ba_4_PrMn_3_O_12_. In 12R-Ba_4_PrMn_3_O_12_, we observe a sharp, likely antiferromagnetic transition at *T*_2_ ≈ 12.15 K and an additional transition at *T*_1_ ≈ 200 K, likely in canted antiferromagnetic order. These results suggest that careful variation of composition within the family of hexagonal, layered perovskites can be used to tune material properties using the complex role of the Pr^4+^ ion in magnetism.

## Introduction

The push for new functional materials to meet ever-evolving societal demands necessitates the continuous discovery and development of materials with desired and tunable properties. Toward this end, the perovskite family and related structures have been a hotbed of material research over the past several decades as a significant portion of the periodic table can form stable *ABX*_3_ perovskites and various more complex motifs.^1−5^*ABX*_3_ perovskites are technologically important in many applications, and often substituting additional cations on the A and/or B sites to form multinary perovskites is an important tool to tune the material’s properties for the desired applications.^6−8^ An opportunity to further expand perovskite functionality exists through layered permutations like hexagonal, perovskite materials that alternate *ABX*_3_ layers and other motifs.^1,9−11^ Within this family, polytypes comprising a multitude of stacking sequences exist. While these materials are more complex than the strictest definitions of perovskites, we refer to these layered, hexagonal perovskite-like structures as perovskites for the purpose of clarity in this work. Many of these structures show complex and varying interactions of the respective polyhedra within the structure, creating a parameter space in which material properties might be tuned by modifying subtle interactions between atoms/polyhedra within the layered structure.^1,11^

**Figure 1:**
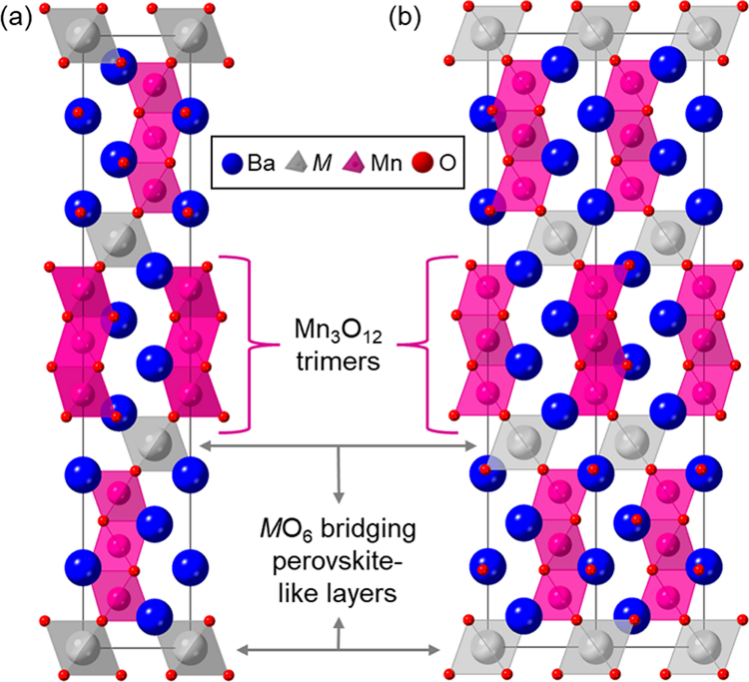
Schematic of the 12R-Ba_4_MMn_3_O_12_ structure aligned to (a) the *a*-axis and (b) the [110] plane.

Of particular recent interest is a subset of the hexagonal, layered perovskite family with the stoichiometry Ba_4_MM′_3_O_12_, in which M= lanthanides, Nb, Ni, and Sn and M′ = Mn, Fe, Ru, and Ir.^1,11−18^ Many members of this family have been investigated for interesting magnetic properties, including frustrated magnetism and possible spin liquid behavior.^11^ They are possible quantum materials due to their mixture of corner-sharing and face-sharing octahedra for the M and M′ sites, respectively (Figure 1 for M′ = Mn), which results in multiple competing magnetic interactions at a range of length scales. Within the Ba_4_MM′_3_O_12_ literature, an interesting example is Ba_1+*x*_CeMn_*x*_O_3+3*x*_, referred to as the BCM family.^19−25^ The BCM family forms three known polytypes, denoted 12R, 10H, and 6H due to rhombohedral (R) and hexagonal (H) symmetry and different number of *a* × *b* planes repeated in the stacking sequence within each unit cell.^21,22,26^ While the 12R polytype is stable at the Ba_4_CeMn_3_O_12_ stoichiometry, as oxygen vacancies are introduced, the 10H and 6H polytypes become the stable phases. In the case of BCM, these oxygen vacancies are charge compensated by Mn^4+^/Mn^3+^ redox behavior at high temperatures, reacting with steam to produce hydrogen or CO_2_ to produce CO.^19,23,26^

The BCM family thus presents a fascinating system to study the relationship between structural complexity and magnetic properties. A particularly interesting feature of this family is the Mn_*x*_O_3+3*x*_ clusters that form from face-sharing MnO_6_ octahedra along the *c*-axis of the unit cell (see Figure 1 for the *x* = 3 or 12R polytype; Mn_3_O_12_ face-sharing clusters are shown in magenta). The clusters are connected via corner-sharing MO_6_ octahedra (Figure 1, gray). The BCM polytypes each have a different number of Mn^4+^ cations within the cluster (dimers in 6H, trimers in 12R, tetramers in 10H) and must also be influenced by the properties of the M cations as well as the possible presence of defects or nonstoichiometry.^1,24^ It is known that clusters of transition metal or lanthanide cations/oxide polyhedra with highly correlated electrons can develop magnetic ordering, depending on the electron filling within the particular structure. Such behavior can be impacted by structural factors such as charge ordering, oxygen stoichiometry, and interactions with neighboring ions/polyhedra. Cluster behavior can emerge in these trimers due, in part, to the short M′–M′ distances (∼2.5 Å for 12R-BCM, for example). In the 12R structure family, cluster behavior has been observed for cases of M = Nb, Sn, and lanthanides with M′ = Mn, as well as for analogues where M′ = Ru, Ir.^12−15,17,27,28^ How cluster behavior among M′ is related to the bridging M cation is also poorly understood.

The presence of nonmagnetic Ce^4+^ as the bridging cation makes 12R-BCM an excellent reference case for understanding the broader role of the M bridging cation on the magnetic behavior of 12R structures, and in a broader sense on hexagonal perovskites.^20−22^ Macias et al. reported the magnetic properties of 12R-BCM, showing a transition to long-range antiferromagnetic (AFM) order at *T*_N_ = 6 K with an anomalously large Weiss temperature (Θ < −2000 K) and an effective moment much lower than the expected value for three *S* = 3/2 Mn^4+^ cations.^21^ The suppressed moment supports possible cluster magnetism in the 12R-BCM Mn_3_O_12_ trimers, but this hypothesis was not explored further. Prior analysis was hampered by the low purity of the samples as well as the presence of nearly equivalent concentrations of Mn-containing secondary phases.

We focus here on 12R-Ba_4_MMn_3_O_12_ and explore the effect(s) of replacing nonmagnetic M = Ce^4+^ with magnetic Pr^4+^. Comparing Ce^4+^ and Pr^4+^ structural analogues is apt because Ce^4+^ and Pr^4+^ have nearly identical ionic radii, are isovalent, and form isostructural 12R materials.^20^ The lone f electron of Pr^4+^ (*J*_eff_ = 1/2) often leads to complex magnetic behavior or unique correlated phenomena;^29^ we hypothesize that when Pr^4+^ is the bridging cation (M) in the 12R polytype, it may facilitate coupling effects/ordering of Mn_3_O_12_ trimers and therefore affect the overall material’s magnetic properties. However, the magnetic properties of 12R-BPM have not been reported.

In this work, we embark on a higher precision examination of the magnetic and thermodynamic properties of 12R-BCM and study 12R-BPM for the first time. We synthesize high phase purity 12R-BCM and 12R-BPM via established methods^20,25^ and study their crystal structures using Rietveld refinements of synchrotron powder X-ray diffraction (PXRD) data. Magnetic and thermodynamic properties are investigated via DC magnetization, AC susceptibility, and heat capacity measurements. While we confirm that 12R-BCM is an AFM with *T*_N_ ≈ 7.5 K and cluster magnetism within its Mn_3_O_12_ trimers, 12R-BPM displays three magnetic transitions and complex behavior. Both materials exhibit indications of magnetic frustration. Our isostructural comparison of 12R-BCM and 12R-BPM highlights the importance of the role of the M cation bridging the Mn_3_O_12_ trimers in the magnetic properties of this polytype.

## Experimental Section

### Synthesis

Synthesis of 12R-BCM was performed by an established solid-state method.^20,25^ BaCO_3_ (99.999%, Sigma-Aldrich) and Ce_2_(CO_3_)_3_·H_2_O (99.9%, Sigma-Aldrich) precursor powders were first dried while MnO_2_ (99.9%, Alfa Aesar) was used as-received. Stoichiometric amounts of each precursor powder were ground with an agate mortar and pestle prior to weighing and mixing. The precursor mixture was loaded into a 1″ diameter steel die and compacted into a pellet. A continuous force of 750 lbs. was applied while the press chamber was evacuated, prepressing the sample to remove air pockets. Force was then ramped to 17,000 lbs. at a rate of 1000 lbs/min, held for 15 min, and then force was released at the same rate, returning to 750 lbs. of force. The pressing environment was held at ≈10 °C and a vacuum of 1.0^–4^ mTorr or below. The resulting green pellet was then fired at 950 °C in air to decompose the carbonates. The sample was loaded into a tube furnace held at 300 °C in a Pt-foil lined alumina crucible and ramped to the desired temperature at 5 °C/min, held at the desired temperature for 20 h, and then returned to 300 °C at the same ramp rate. After firing, the sample was ground by hand using an agate mortar and pestle. Three additional press-fire-grind iterations were performed with the first featuring a firing temperature of 1350 °C and the final two iterations using a firing temperature of 1500 °C.

12R-BPM was synthesized by adapting a Pechini sol–gel method that was previously demonstrated for 12R-BCM.^20,25^ All chemicals were purchased and used as-received: Ba(NO_3_)_2_ (Alfa Aesar ACS, 99+%), Pr(NO_3_)_3_·6H_2_O (99.9%, Aldrich), Mn(NO_3_)_2_·4H_2_O (98%, Alfa Aesar), and anhydrous citric acid (Fisher Scientific, certified ACS). The cation precursors and anhydrous citric acid at a 1:1.5 mol ratio was first dissolved in deionized water (∼125 mL for ∼5 g product) and stirred on a heated hot plate until most of the water had evaporated, leaving a viscous liquid. The resulting liquid was dried overnight at 110 °C in air, yielding a foam-like solid. This solid was ground into a powder and then self-combusted on a hot plate in air, after which the resulting solid was again ground and transferred to an alumina crucible for high-temperature processing. A subsequent calcination at 800 °C (ramped at 5 °C/min) in air was performed for a duration of 12 h to remove any remaining organics or precursor ligand residue. The calcined powder was reground, and then repeatedly sintered with intermittent grinding at 1250 °C (ramped at 10 °C/min) in air for 12 h over a total of 4 grinding/heating iterations, as adapted from the literature.^20^

### Characterization

Synchrotron PXRD measurements were performed at Stanford Synchrotron Radiation Lightsource (SSRL) beamline 2–1, employing an incident wavelength of 0.729 Å (17.0 keV). Powder samples were flame-sealed in 0.5 mm glass capillaries purchased from Charles Supper Company. Measurements were performed in a Debye–Scherrer geometry with capillaries spinning at approximately three rotations per second. Angle-resolved scans were obtained between 5 and 115° 2θ with 1° steps using a Pilatus 100 K area detector with a 700 mm sample-to-detector distance. Two-dimensional (2D) images were normalized to the incident beam current and subsequently integrated using a Python script developed at SSRL. Rietveld refinements were performed on the XRD data using TOPAS-academic.^30^ An Oxford Instruments Cryojet was used to cool the sample capillaries to 100 K. The data collected at 100 K was deposited into the ICSD for both 12R-BCM (Deposition Number 2328392) and 12R-BPM (Deposition Number 2307846).

DC magnetization and AC susceptibility measurements were performed in a Quantum Design Physical Properties Measurement System (PPMS) from *T* = 2–350 K in applied fields up to μ_0_*H* = 14 T. AC susceptibility was measured with a 1.5 Oe drive current at frequencies greater than 1500 Hz and with a 5 Oe drive current at frequencies lower than 1500 Hz. Heat capacity measurements were performed in a different PPMS on pressed pellets of powder in applied fields up to μ_0_*H* = 9 T. Apiezon N grease was used to adhere the pellet to the puck. Three data points were collected at each temperature; the value used was the average of the second and third data points. A background was fit empirically to *C*_bg_ = *aT*^3^ + *bT*^2^ between *T* = 12 and 20 K for 12R-BCM in the nonzero field (*T* = 12–30 K for μ_0_*H* = 0 T) and between *T* = 17.5 and 30 K for 12R-BPM in nonzero field (*T* = 12–35 K for μ_0_*H* = 0 T).

## Results

### Synthesis and Structure

**Figure 2:**
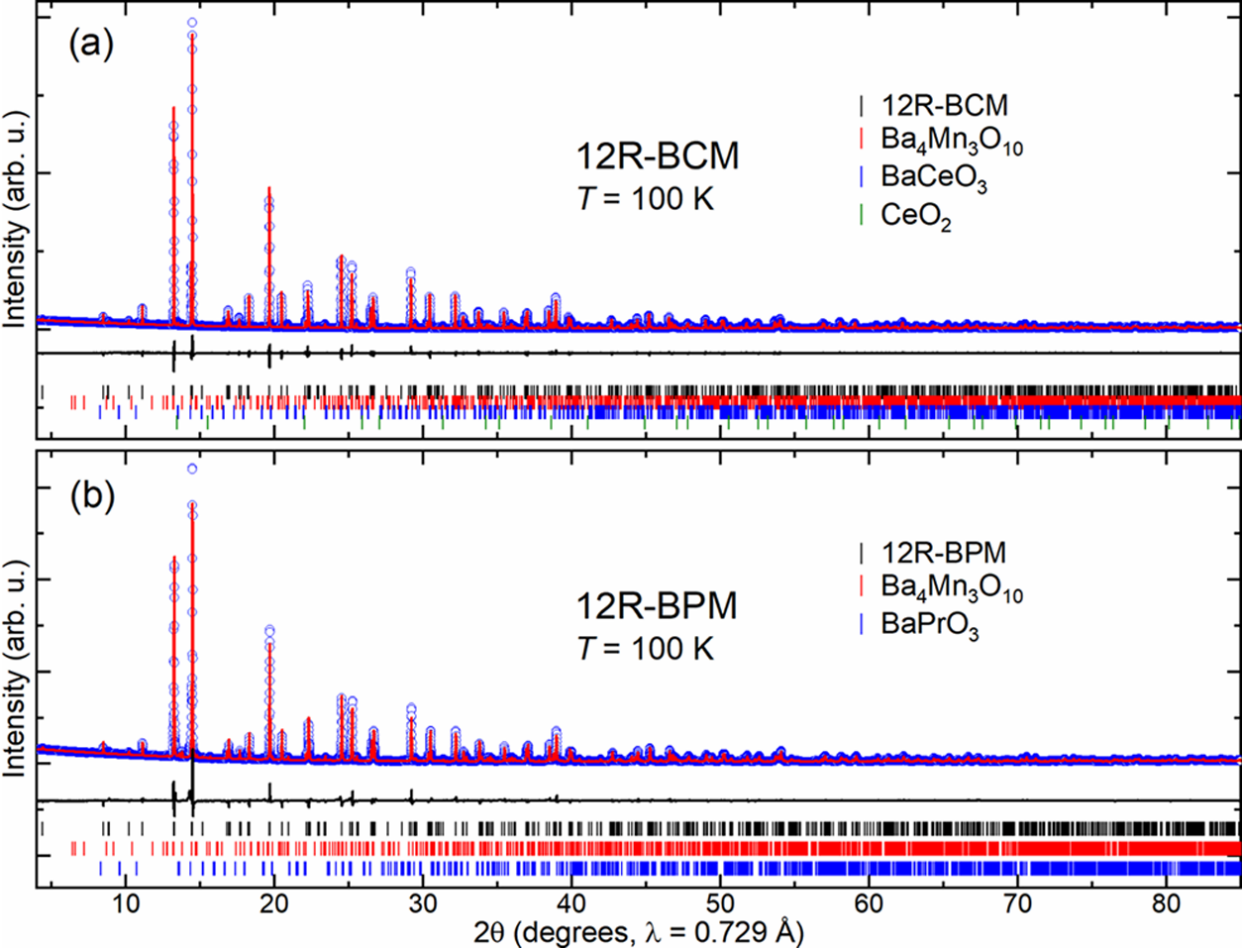
Rietveld refinements of synchrotron PXRD data collected at 100 K for (a) 12R-BCM and (b) 12R-BPM. Observed (blue), calculated (red), and difference (black) plots are shown, and Bragg reflections are indicated by tick marks.

To explore the extent of structural changes resulting from replacing Ce^4+^ for Pr^4+^ in 12R-BCM, synchrotron PXRD data were collected at *T* = 100 K (Figure 2) and *T* = 300 K (Figure S1) for both materials. Rietveld refinements show that both samples are primarily comprised of the 12R structure, with lattice parameters matching prior structural reports in the literature,^20,25^ as displayed in Figure S1 and Tables S1–S2. The structures of the Ce and Pr analogues are nearly identical, with 12R-BCM only ∼0.25% larger in the in-plane (*ab*) direction and ∼0.14% larger along the *c*-axis compared to 12R-BPM. No 6H or 10H structural impurity is observed for either sample.

Comparing the low- and room-temperature PXRD data (Figures 2 and S1) indicates that for both materials, no structural differences or symmetry lowering are present between 100 and 300 K, beyond anisotropic thermal expansion similar to that observed at elevated temperatures for 12R-BCM.^25^ Lattice parameters and refinement details are listed in Table S1. Comparing the lattice parameters of 12R-BPM at 100 and 300 K reveals anisotropic lattice contraction (0.0018% in-plane and 0.0014% out-of-plane), implying that upon cooling, the Mn_3_O_12_ trimers become proportionally closer in the *ab* plane than along the *c*-axis.

A summary of the weight fractions of each phase identified via Rietveld refinement is presented in Table S2, confirming the high phase purity of 12R-BCM and 12R-BPM. This sample of 12R-BCM has a phase purity of 98.39 ± 0.1 wt %, consistent with previous reports using the same synthesis.^25^ Very small (<1 wt %) amounts of impurity phases Ba_4_Mn_3_O_10_, BaCeO_3_, and CeO_2_ were identified via Rietveld refinement. In comparison to our 98.39 ± 0.1 wt % 12R-BCM sample, the prior magnetic investigation in the literature used 11–31 wt % 12R-BCM.^21^ Our samples possess no measurable quantity of either Ba_6_Mn_5_O_16_ or BaMnO_3_, whereas in comparison, the previous magnetic report of 12R-BCM possessed these impurities at levels of >11.5 and >5.5 wt %, respectively.^21^ By removing these magnetically active impurities, a higher confidence is achieved in isolating the behavior of 12R-BCM. The 12R-BPM sample studied here similarly has very high phase purity of 96.6 ± 0.5 wt %, with small impurity phases of Ba_4_Mn_3_O_10_ and BaPrO_3_. While both impurities found in our 12R-BPM sample are magnetic, the very low fraction of these impurities limits signals from their magnetic behaviors from contributing to our magnetic results at detectable levels, as discussed below.

### Magnetic Properties

**Figure 3:**
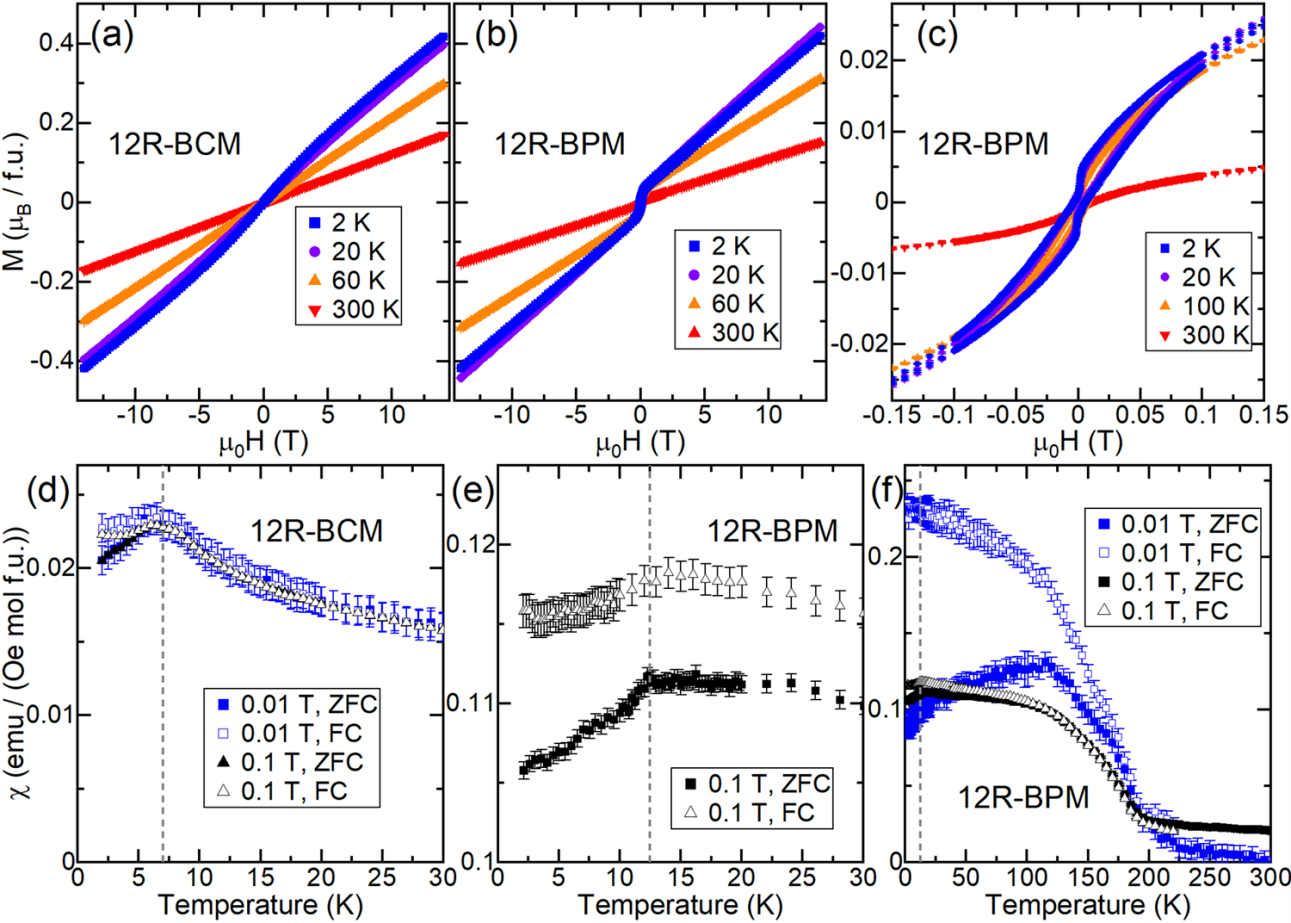
(a, b) Magnetization (M) as a function of applied field for (a) 12R-BCM and (b) 12R-BPM. (c) Magnified low-field view of M vs applied field for 12R-BPM. (d) DC susceptibility (χ) of 12R-BCM in low applied fields. (e, f) χ of 12R-BPM in low applied fields. The dashed lines denote the approximate position of each material’s low-temperature transition. ZFC signifies zero-field-cooled, and FC signifies field-cooled.

To probe the effects of the M^4+^ cation on the magnetism of these materials, we measured the DC magnetization (M ) of polycrystalline samples of both 12R-BCM and 12R-BPM as a function of temperature and the applied field. Figure 3(a,b) shows isothermal field sweeps performed at several temperatures for each sample. The 12R-BCM data are consistent with the reported long-range antiferromagnetic (AFM) order (Figure 3a);^21^ there is no hysteresis or net moment at *T* = 2 K. In contrast, at low temperature, 12R-BPM (Figure 3b,c) displays hysteresis with a small net moment and a distinctive wasp-waisted appearance that persists at least up to *T* = 100 K and is gone at *T* = 300 K. A comparison of the low-field regions of the two materials is given in Figure S2, confirming that 12R-BCM has no discernible net moment or hysteresis.

Zero-field-cooled (ZFC) and field-cooled (FC) DC susceptibility (χ) as a function of temperature at low applied fields is shown in Figure 3d–f for both samples. 12R-BCM displays a peak at approximately *T* ≈ 7 K (Figure 3d), consistent with the *T*_N_ = 6 K reported previously.^21^ In 12R-BCM, a small amount of splitting below the *T*_N_ is observed for the first time (Figure 3d). Variable-frequency AC susceptibility measurements (Figures S3–S4) of 12R-BCM show a peak in the real part (χ′) but no frequency dependence, consistent with AFM order and suggesting that the ZFC-FC splitting is more likely due to sample disorder than to a spin glass state.

**Figure 4:**
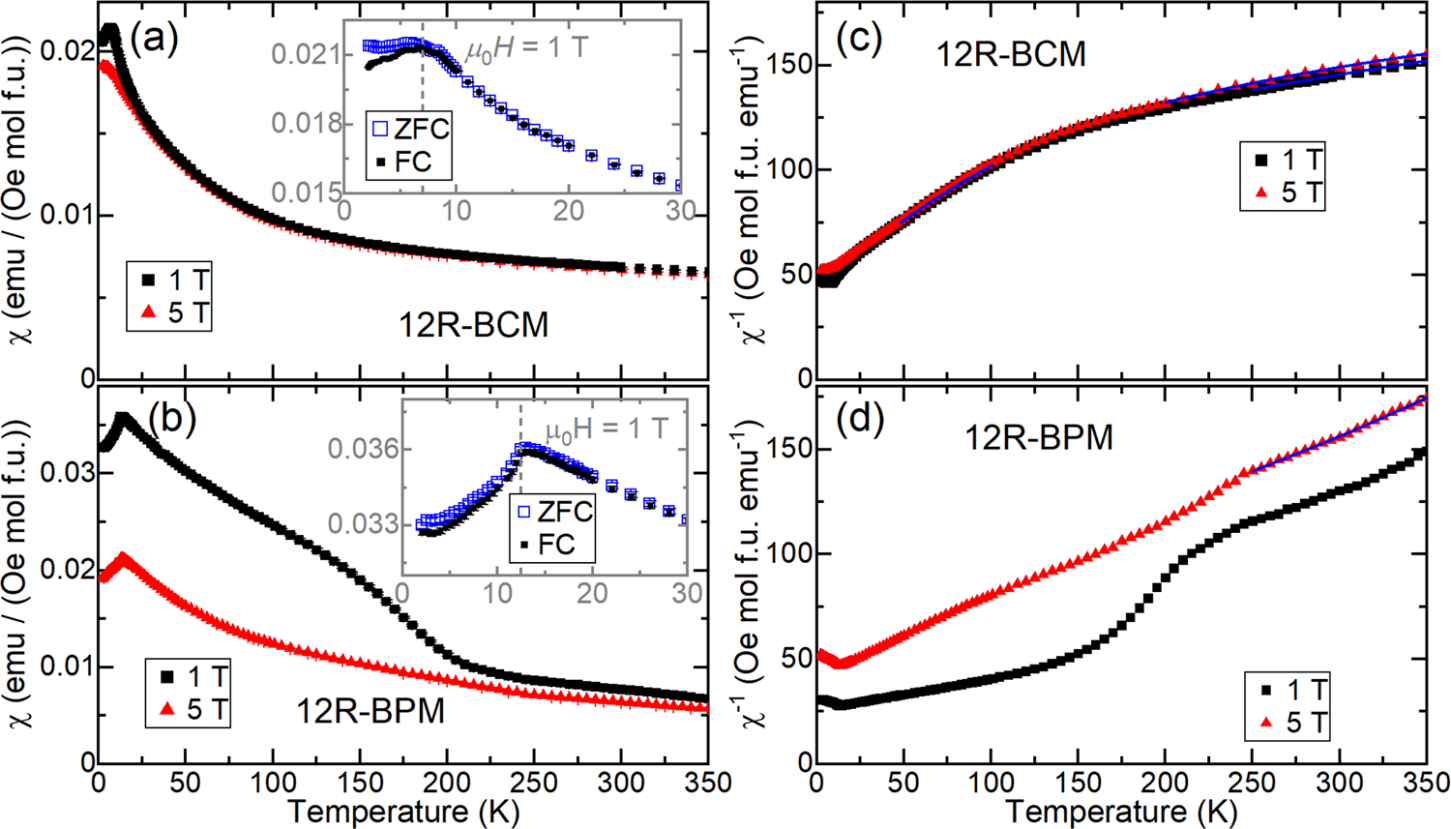
(a, b) DC susceptibility (χ) of (a) 12R-BCM and (b) 12R-BPM collected in several applied fields. The insets show the ZFC and FC data collected at μ_0_*H* = 1 T; the dashed lines denote the approximate position of each material’s low-temperature transition. (c, d) Inverse susceptibility data and Curie–Weiss fits (blue lines) for (c) 12R-BCM and (d) 12R-BPM.

The magnetic behavior of the 12R-BPM sample is more complex than that of 12R-BCM. Like 12R-BCM, the 12R-BPM susceptibility data display a peak that appears consistent with AFM order, albeit at *T* ≈ 12.5 K (Figures 3e and 4b), which is ∼5.5 K higher than the peak observed for 12R-BCM. Further evidence for AFM order in 12R-BPM is the lack of a significant change in the field-dependent magnetization on either side of this peak (see Figure 3c, *T* = 2 and 20 K). However, it is apparent in the μ_0_*H* = 0.1 T data of 12R-BPM (Figure 3e) that ZFC-FC splitting exists above the peak at *T* ≈ 12.5 K. To probe this ZFC-FC splitting further, we measured up to *T* = 300 K in several applied fields, as shown in Figure 3f. This reveals an additional transition occurring for 12R-BPM at approximately *T*_1_ ≈ 200 K, with significant ZFC-FC splitting beneath it. The presence of hysteresis and a small net moment below this transition, coupled with the absence of atomic rearrangement confirmed by XRD, suggests that below this, ≈200 K transition is either a weak ferrimagnetic or a canted AFM state. These potential ground states are consistent with the wasp-waisted appearance of the magnetization curves (Figure 3c), as wasp-waisted behavior is thought to arise from competition between AFM and FM ground states.^21^

While several impurities found in our 12R-BCM and 12R-BPM samples are magnetic, these phases are only present at very low phase fractions (see Table S2), and we do not observe peaks at their respective ordering temperatures in our susceptibility data (Ba_4_Mn_3_O_10_ is reported AFM below *T*_N_ ≈ 40 K; BaPrO_3_ is AFM below *T*_N_ ≈ 11.7 K),^31,32^ confirming that the observed magnetic behavior contains very little contribution from magnetic secondary phases.

DC susceptibility data collected at higher applied fields are listed in Figure 4. For 12R-BCM (Figure 4a), the data have a peak at *T*_N_ ≈ 7 K, and the 1 and 5 T data sets are coincident above *T* ≈ 50 K. A small amount of ZFC-FC splitting is evident at μ_0_*H* = 1 T below *T*_N_ (Figure 4a, inset). Analogous data were collected for 12R-BPM, as shown in Figure 4b, where the transition at *T*_2_ ≈ 12.5 K is evident, and no ZFC-FC splitting is observed at μ_0_*H* = 1 T (Figure 4b, inset). 12R-BPM’s transition at *T*_1_ ≈ 200 K is visible in the μ_0_*H* = 1 T data but suppressed in the μ_0_*H* = 5 T data. Above this transition, 12R-BPM’s 1 and 5 T data sets are mostly coincident.

Curie–Weiss fits of the high-temperature (*T* = 250–350 K) inverse susceptibility data (Figure 4c,d) were performed for both samples with diamagnetic corrections (χ_0_, see Table 1). This temperature range was chosen to be above the *T* ≈ 200 K transition of 12R-BPM; however, we note that the 12R-BPM inverse susceptibility data in this range are still not linear, implying that fits for 12R-BPM may not meaningfully represent the paramagnetic regime. The extracted parameters are given in Table 1; unfortunately, the small fit range led to large uncertainties in some cases. The extracted Weiss temperatures (Θ) are large and negative for both samples, indicating strong AFM interactions. The Θ extracted for 12R-BCM is approximately −130 to −220 K, an order of magnitude lower than the previously reported value of −2000 K, which was attributed to short-range interactions persisting up to near-room temperature.^21^ We posit that our extracted values are more reflective of the intrinsic behavior of 12R-BCM due to the much higher phase purity of our sample. Compared to 12R-BCM, 12R-BPM exhibits an even larger Θ ≈ −520 K, indicating even stronger AFM correlations. Nevertheless, comparing these Θ values to the low Neél temperatures suggests the presence of strong magnetic frustration in both 12R-BCM and 12R-BPM.

**Table 1:** Parameters Extracted from Curie–Weiss Fits^a^

	12R-BCM	12R-BCM	12R-BCM	12R-BPM
temp. fit range (K)	47–100	250–350	250–350	250–350
applied field (T)	1	1	5	5
*C* (K emu mol^–1^)	0.9(0.05)	1.8(0.5)	1.17(0.7)	9.6(4.6)
Θ (K)	–42(3)	–223(70)	–128(120)	–522(197)
μ_eff, f.u._ (μ_B_)	2.65(0.07)	3.8(0.5)	3.1(0.9)	9(2)
μ_eff, Mn_ (μ_B_)	1.53(0.04)	2.2(0.3)	1.8(0.5)	---
χ_0_ (emu Oe^1–^ mol^–1^)	3.63 × 10^–3^	3.48 × 10^–3^	3.98 × 10^–3^	–5.33 × 10^–3^

a Fit uncertainties are given in parentheses.

The effective magnetic moment per formula units (μ_eff,f.u._) extracted from Curie–Weiss fits of 12R-BCM (see Table 1) are low compared to the expected value for three independent *S* = 3/2 Mn^4+^ cations (6.71 μ_B_). The fact that our fits give an average value of ∼3.4 μ_B_ is indicative of correlations between the magnetic cations, consistent with the structural motif of the Mn_3_O_12_ clusters. Indeed, our extracted value (∼3.4 μ_B_) is closer to the expected value for one Mn^4+^ (∼3.9 μ_B_). Upon assuming simple AFM alignment, if the Mn_3_O_12_ motifs of 12R-BCM form magnetic clusters, then the net moment per formula unit should be approximately *S* = 3/2, equivalent to one Mn^4+^ cation. Our data are consistent with this hypothesis.

We note there are two linear regions of the 12R-BCM inverse susceptibility data in Figure 4c—roughly 50 < *T* < 100 K and 200 < *T* < 350 K—suggesting that different behavior is occurring in these temperature ranges, such as short-range correlations below *T* ≈ 150 K. Indeed, a Curie–Weiss fit of the 12R-BCM data in the lower temperature linear region (from 47–100 K, Table 1) yields an even lower μ_eff,f.u._ than in the higher-temperature region, consistent with strengthening of intertrimer correlations and short-range ordering.

For 12R-BPM, we used Curie–Weiss fitting to determine a μ_eff,f.u._ but did not extract a μ_eff,Mn_ from this fit due to the presence of both Mn^4+^ and Pr^4+^ in the material. The μ_eff,f.u._ of 12R-BPM is significantly higher than that of 12R-BCM, consistent with the addition of Pr^4+^ (*J*_eff_ = 1/2). The extracted value (9 μ_B_) is within error of the calculated value based on a free-ion model of three Mn^4+^ cations and one *J*_eff_ = 1/2 Pr^4+^ (7.2 μ_B_). The large error is likely due to the fitted data not being fully paramagnetic, as discussed above, and the small fit range. Interestingly, 12R-BPM does not appear to exhibit the same suppression in the effective moment that 12R-BCM does, possibly reflecting fewer short-range correlations in this temperature range compared to 12R-BCM.

### Thermodynamic Properties

**Figure 5:**
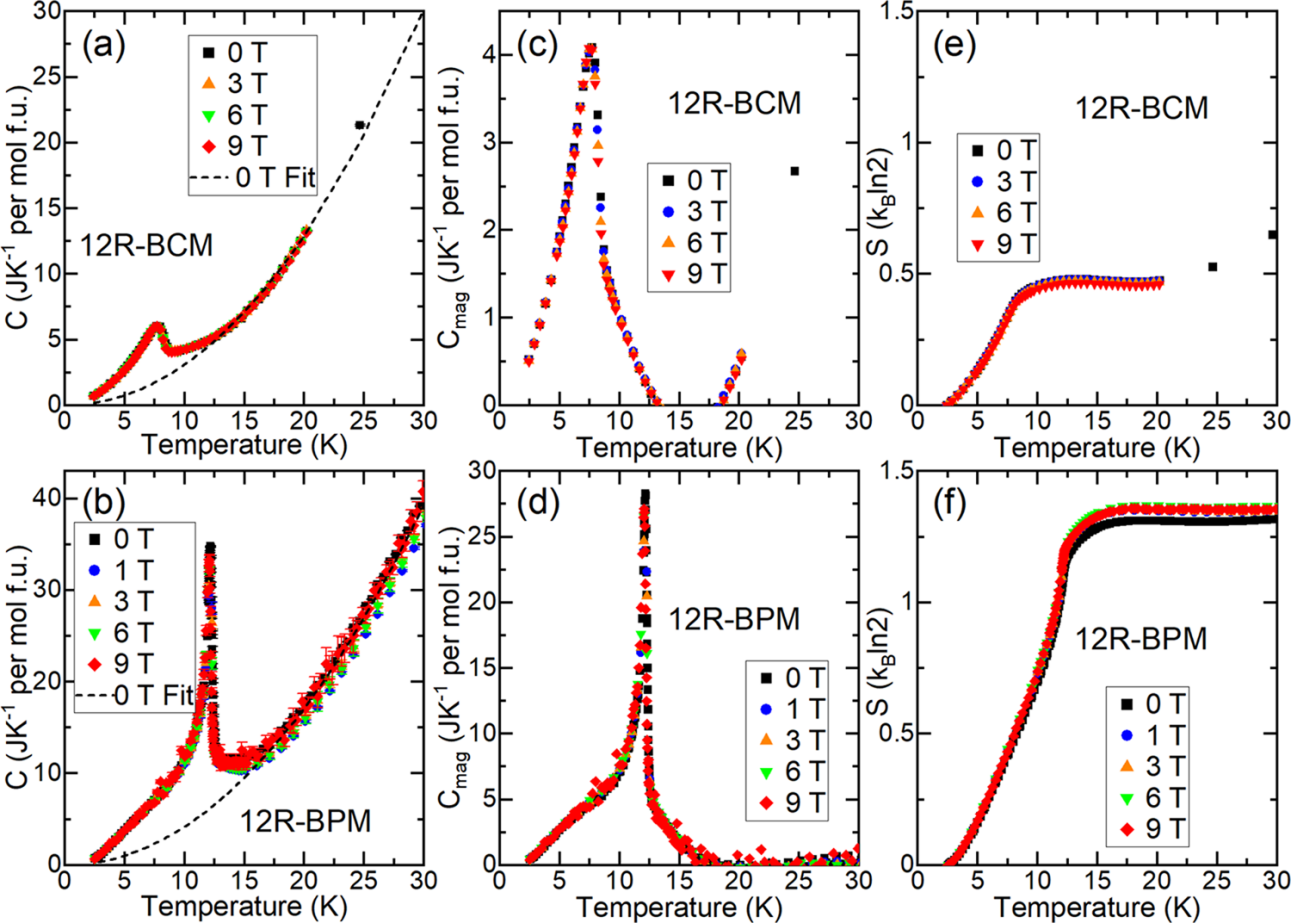
Heat capacities of 12R-BCM and 12R-BPM. (a, b) Molar *C*_p_; the dashed lines indicate the background (*C*_bg_) for each zero-field curve extrapolated to *T* = 0 K. (c, d) *C*_mag_ calculated by subtracting *C*_bg_ from the molar *C*_p_. (e, f) Magnetic entropy normalized per formula unit.

To examine the entropy changes associated with the low-temperature transitions, heat capacity measurements were collected on powder samples of 12R-BCM and 12R-BPM in applied fields from 0–9 T. The molar heat capacity (*C*_p_, Figure 5a) shows that 12R-BCM has a λ peak at *T*_N_ = 7.75 K, very close to the peak observed in the magnetization measurements. For 12R-BPM (Figure 5b), a sharp λ peak at *T*_2_ = 12.15 K is observed, in reasonable agreement with the *T*_2_ ≈ 12.5 K transition in the susceptibility data and consistent with long-range AFM order and the lower transition observed in the susceptibility data. The peak in the 12R-BPM heat capacity data is much larger per mole than for 12R-BCM. No significant changes in the amplitude of either material’s peak were observed in applied fields up to μ_0_*H* = 9 T for either material (Figure 5a,b). However, the peak positions shift to lower temperatures by approximately 0.1 K in an applied field of μ_0_*H* = 9 T for both materials, as shown in Figure S6. This confirms that the origins of these peaks are magnetic.

The background of each *C*_p_ curve was fit empirically to *C*_bg_ = *aT*^3^ + *bT*^2^; the zero-field fits are shown in Figure 5a,b. The heat capacity related to the magnetic transition(s) (*C*_mag_) shown in Figure 5c,d was calculated by subtracting the background (*C*_bg_) from *C*_p_. The onsets of the transitions are relatively sharp in both materials, although the *C*_mag_ of 12R-BPM at *T*_2_ ≈ 12.15 K is approximately six times higher than that of 12R-BCM at *T*_N_ = 7.75 K, suggesting that Pr^4+^ plays an important role in this *T*_2_ ≈ 12.15 K magnetic transition. Additionally, at low temperatures, the *C*_p_ of 12R-BPM exhibits a wide shoulder that resembles the 12R-BCM peak at 7.75 K.

We calculated the entropy (*S*) per formula unit released by these transitions (Figure 5e,f) by integrating *C*_mag_/*T* as a function of the temperature to yield the entropy released by these transitions. As the magnetic cations are complex, the data were normalized per formula unit rather than per magnetic cation. The entropy released by the transition in 12R-BCM plateaus at approximately 0.5 *k*_B_ ln(2), a relatively low value that is consistent with cluster magnetism. The entropy released by the low-temperature transition(s) of 12R-BPM plateaus at approximately 1.3 *k*_B_ ln(2), reflecting a large contribution of the Pr^4+^ cation to the low-temperature magnetic behavior.

To investigate the transition at *T*_1_ ≈ 200 K observed in the 12R-BPM susceptibility data, we measured *C*_p_ up to approximately *T* = 250 K in several applied fields for both materials, as shown in Figure S7. While a slight anomaly is observed at *T* ≈ 220 K in 12R-BPM, a similar anomaly is also visible in the 12R-BCM data, despite the 12R-BCM sample having no noticeable transition near that temperature in the magnetic data. The appearance of this anomaly near 220 K for both samples suggests that this is likely due to instrumental or sample mounting artifacts instead sample behaviors. The lack of a signature in the heat capacity implies that the *T*_1_ transition in 12R-BPM is relatively weak and is consistent with the XRD data showing that it is not related to a structural transition.

## Discussion

Although 12R-BCM and 12R-BPM are isostructural down to 100 K, 12R-BPM exhibits a magnetic transition at *T*_1_ ≈ 200 K—which has no analogue in 12R-BCM—and at least one additional low-temperature transition (*T*_2_ ≈ 12.15 K). Below the 12R-BPM *T*_1_ transition, we observe a small net moment on the order of 0.05 μ_B_ per f.u. and hysteresis with a characteristic wasp-waisted appearance (Figure 3c). This suggests that the order is likely canted AFM or ferrimagnetism. The large, negative Weiss temperature supports that the overall correlations are AFM. We rule out a structural phase transition near this *T*_1_ transition using the 100 and 300 K synchrotron PXRD data (Figure 2), which show no signs of symmetry changes. In addition, there was no hysteresis when we measured magnetization while warming and cooling through this temperature (Figure S5) and no peak in the heat capacity at this temperature (Figure S7). Instead, our results indicate that this *T*_1_ transition in 12R-BPM is a purely magnetic or electronic transition. The wide temperature regime over which this transition occurs may indicate some heterogeneity in the 12R-BPM sample.

While the most significant differences between 12R-BCM and 12R-BPM are attributed to the substitution of Pr^4+^ for Ce^4+^, our results may additionally be consistent with a hypothesis that 12R-BPM’s *T*_1_ ≈ 200 K transition is caused by a small amount of oxygen vacancies (below 1%). Oxygen vacancies could cause some reduction of Pr^4+^ or Mn^4+^ to Pr^3+^ or Mn^3+^. The formation of oxygen vacancies in 12R-BCM, thought to be charge compensated by Mn^4+^ to Mn^3+^ transitions, and the influence of oxygen vacancies on phase stability have been reported previously.^26^ To combat oxygen-vacancy formation, our 12R-BCM and 12R-BPM samples were thoroughly annealed in air, under which conditions we expect negligible quantities of oxygen vacancies.

The 150–200 K behavior of both 12R-BCM and 12R-BPM is consistent with many previous reports of non-Curie–Weiss behavior of Mn^4+^ cations near-room temperature.^33,34^ Our assignment of the *T*_1_ transition in 12R-BPM as arising from Mn instead of Pr is supported by the short-range ordering of Mn at a similar temperature in 12R-BCM (∼150 K) as well as the observed ferrimagnetic order at 42 K of the Nb^5+^ analogue, Ba_4_NbMn_3_O_12_, whose trimers are composed of Mn^3+^Mn_2_^4+^O_12_, yielding a net cluster moment of *S* = 2.^12^ In addition, the *T*_1_ transition may be influenced by oxygen-vacancy-induced reduction of Mn^4+^ to Mn^3+^, although this effect should be small since the oxygen vacancies in these samples are expected to be near dopant levels. The role of partial reduction on the M′ site of 12R materials is not well described by theory, but many examples exist in the literature that are related to what we observe here. A similar onset in the magnetization at approximately *T* ≈ 200 K was observed in isostructural 12R material Ba_4_Ni^4+^Fe^3+^Fe_2_^4+^O_11.5_, in which one highly oxidized Fe^4+^ is reduced to Fe^3+^; this onset was attributed to interactions between the mixed valent Fe ions.^28^ However, given that 12R-BCM does not display this transition at 200 K, we cannot rule out the possibility that the presence of Pr^4+^ is at least in part responsible for this behavior. Further work elucidating the magnetic structure and definitively studying the role of Pr^4+^ or oxygen vacancies in the observed transition will be essential to understanding this transition.

Due to the presence of Pr^4+^, the low-temperature magnetic behavior—and ground state—of 12R-BPM is more complex than the AFM order of 12R-BCM at *T*_N_ ≈ 7.75 K. Given that the *T*_2_ ≈ 12.15 K feature in 12R-BPM releases ∼3× as much magnetic entropy (and occurs about 5.5 K higher) than the 12R-BCM’s AFM transition at *T*_N_ ≈ 7.75 K, the 12R-BPM *T*_2_ transition must involve the Pr^4+^ cations in addition to the Mn_3_O_12_ trimers. There is no significant effect on the net magnetic moment of 12R-BPM due to this *T*_2_ transition, suggesting that it has AFM character, consistent with the extracted Weiss temperature. Another feature may be present in the heat capacity data of 12R-BPM: *C*_p_ and *C*_mag_ display a shoulder below the peak at *T*_2_ ≈ 12.5 K (Figure 5b,d). This shoulder is similar in magnitude to (and present over the same temperature range as) the sole peak present for 12R-BCM at *T*_N_ ≈ 7.75 K, and a direct comparison to illustrate this point is shown in Figure S8. The 12R-BPM heat capacity data could therefore be interpreted as a superposition of behavior strongly shared with 12R-BCM plus a stronger peak associated with both the Pr and Mn sublattices. This interpretation suggests that behavior similar to that occurring through the AFM transition in 12R-BCM, which is likely associated with intratrimer ordering, may also be occurring in 12R-BPM. Altogether for 12R-BPM, we conjecture that at *T*_1_ ≈ 200 K, canted AFM intertrimer order develops; then, at *T*_2_ ≈ 12.15 K, the Mn_3_O_12_ trimers and Pr^4+^ cations order antiferromagnetically; and finally, full intratrimer and Pr^4+^ AFM order occurs at *T*_3_ ≈ 7.5 K. However, neutron diffraction data will be necessary to fully understand the magnetic structures of these materials and validate these hypotheses.

Substituting the bridging nonmagnetic Ce^4+^ cation between the Mn_3_O_12_ trimers with Pr^4+^ (*J*_eff_ = 1/2) also has a significant effect on the cluster magnetism of these materials. In 12R-BCM, we observe an onset of short-range AFM correlations at *T* ≈ 150 K and full AFM order at *T*_N_ ≈ 7.75 K. That the Mn_3_O_12_ trimers in 12R-BCM exhibit cluster magnetism is supported by the results of Curie–Weiss fitting, which yielded an effective moment much smaller than that expected for three Mn^4+^ cations. Instead, it was much closer to the value for *S* = 3/2 per formula unit, which is the net spin expected for this cluster assuming AFM interactions. Our observation of likely cluster magnetism in 12R-BCM is consistent with observations of cluster magnetism in isostructural 12R-Ba_4_NbMn_3_O_12_ and analogues with Mn replaced by Ru or Ir.^12,14,15^ Interestingly, the effective moment of 12R-BPM is within error of the calculated value for three Mn^4+^ cations and a Pr^4+^, suggesting that different behavior may be occurring compared to 12R-BCM. Perhaps the Mn_3_O_12_ trimers in 12R-BPM exhibit less cluster-like behavior due to interactions between Pr^4+^ and these trimers, and/or a small amount of Pr^3+^ or Mn^3+^ defects might be present, both of which could raise the total effective moment.

Although their magnetic structures are necessarily different, the long-range intratrimer order at low temperature in both materials must be mediated by the M^4+^O_6_ octahedra, which are corner-sharing with six Mn_3_O_12_ trimers; in turn, each Mn_3_O_12_ trimer is corner-sharing with six M^4+^O_6_ octahedra—three at each end of the trimer. This plethora of competing magnetic interactions is a likely source of the magnetic frustration suggested by the large ratio of the Weiss temperature to the Neél temperature for both materials (∼37 for 12R-BCM and ∼41 for 12R-BPM).

## Conclusions

We have studied the crystal structures and magnetic properties of two members of a family of hexagonal perovskites in the 12R structure, Ba_4_CeMn_3_O_12_ and Ba_4_PrMn_3_O_12_, where the Ce/Pr site bridges the *c*-axis-aligned trimers of face-sharing MnO_6_ octahedra. While the two materials are isostructural, susceptibility and heat capacity measurements reveal the striking influence of composition on resulting properties. 12R-BCM is a frustrated AFM below *T*_N_ ≈ 7.75 K, and the Mn_3_O_12_ trimers exhibit cluster magnetism. We show for the first time that the substitution of Pr^4+^ (f^1^) for nonmagnetic Ce^4+^ strongly influences the magnetic properties of 12R-BPM. 12R-BPM exhibits three magnetic transitions: the first, at *T*_1_ ≈ 200 K, results in a small net moment and is likely a canted AFM or a ferrimagnetic state. We suggest that this transition may be related to the known non-Curie–Weiss behavior of Mn^4+^ near-room temperature. The main low-temperature transition occurs at *T*_2_ ≈ 12.15 K, which is ∼5.5 K higher than the main 12R-BCM transition; we conjecture that this is related to the AFM ordering of both the Pr and Mn_3_O_12_ trimer sublattices. The f electron contributed by Pr^4+^ likely increases the degree of intratrimer interactions as well as being an additional magnetic cation within the structure. Finally, there may be a weak additional transition at *T*_3_ ≈ 7.5 K related to full intratrimer AFM order. Our data suggest that both 12R-BCM and 12R-BPM are highly frustrated, likely due to the complexity of these structures coupled with the unique magnetic behavior of the Pr^4+^ ion. While full understanding of the subtle magnetic transitions observed in this work will require a further, in-depth study of their magnetic structures, including with neutron diffraction/scattering, our results demonstrate that a rich field of material design awaits within layered, hexagonal perovskite materials.

## Supporting Information Available

Additional crystallographic details, magnetic data, and heat capacity data (PDF)
12R-BCM_100K (CIF)
12R-BPM_100K (CIF)

## Supplementary Material

cm3c03014_si_001.pdf

cm3c03014_si_002.cif

cm3c03014_si_003.cif

## References

[ref1] Tilley R. J. D. Hexagonal Perovskite-Related Structures. In Perovskites: Structure–Property Relationships; Wiley, 2016; pp 79–122.

[ref2] Green M. A.; Ho-Baillie A. Perovskite Solar Cells: The Birth of a New Era in Photovoltaics. ACS Energy Lett. 2017, 2 (4), 822–830. 10.1021/acsenergylett.7b00137.

[ref3] Majher J. D.; Gray M. B.; Strom T. A.; Woodward P. M. Cs_2_NaBiCl_6_:Mn^2+^ — A New Orange-Red Halide Double Perovskite Phosphor. Chem. Mater. 2019, 31 (5), 1738–1744. 10.1021/acs.chemmater.8b05280.

[ref4] Bhalla A. S.; Guo R.; Roy R. The Perovskite Structure—a Review of Its Role in Ceramic Science and Technology. Mater. Res. Innovations 2000, 4 (1), 3–26. 10.1007/s100190000062.

[ref5] Peña M. A.; Fierro J. L. G. Chemical Structures and Performance of Perovskite Oxides. Chem. Rev. 2001, 101 (7), 1981–2018. 10.1021/cr980129f. 11710238

[ref6] Zhou E.; Raulot J.-M.; Xu H.; Hao H.; Shen Z.; Liu H. Structural, Electronic, and Optical Properties of Rare-Earth-Doped SrTiO_3_ Perovskite: A First-Principles Study. Phys. B: Condens. Matter 2022, 643, 414160 10.1016/j.physb.2022.414160.

[ref7] Dzara M. J.; Christ J. M.; Joghee P.; Ngo C.; Cadigan C. A.; Bender G.; Richards R. M.; O’Hayre R.; Pylypenko S. La and Al Co-Doped CaMnO_3_ Perovskite Oxides: From Interplay of Surface Properties to Anion Exchange Membrane Fuel Cell Performance. J. Power Sources 2018, 375, 265–276. 10.1016/j.jpowsour.2017.08.071.

[ref8] Xu L.; Yuan S.; Zeng H.; Song J. A Comprehensive Review of Doping in Perovskite Nanocrystals/Quantum Dots: Evolution of Structure, Electronics, Optics, and Light-Emitting Diodes. Mater. Today Nano 2019, 6, 100036 10.1016/j.mtnano.2019.100036.

[ref9] Lee D.; Lee H. Controlling Oxygen Mobility in Ruddlesden–Popper Oxides. Materials 2017, 10 (4), 368 10.3390/ma10040368. 28772732 PMC5506909

[ref10] Xu X.; Zhong Y.; Shao Z. Double Perovskites in Catalysis, Electrocatalysis, and Photo(Electro)Catalysis. Trends Chem. 2019, 1 (4), 410–424. 10.1016/j.trechm.2019.05.006.

[ref11] Nguyen L. T.; Cava R. J. Hexagonal Perovskites as Quantum Materials. Chem. Rev. 2021, 121 (5), 2935–2965. 10.1021/acs.chemrev.0c00622. 32955868

[ref12] Nguyen L. T.; Kong T.; Cava R. J. Trimers of MnO_6_ Octahedra and Ferrimagnetism of Ba_4_NbMn_3_O_12_. Mater. Res. Express 2019, 6 (5), 056108 10.1088/2053-1591/ab0695.

[ref13] Nguyen L. T.; Halloran T.; Xie W.; Kong T.; Broholm C. L.; Cava R. J. Geometrically Frustrated Trimer-Based Mott Insulator. Phys. Rev. Mater. 2018, 2 (5), 054414 10.1103/PhysRevMaterials.2.054414.

[ref14] Shimoda Y.; Doi Y.; Hinatsu Y.; Ohoyama K. Synthesis, Crystal Structures, and Magnetic Properties of New 12L-Perovskites Ba_4_LnRu_3_O_12_ (Ln = Lanthanides). Chem. Mater. 2008, 20 (13), 4512–4518. 10.1021/cm800708g.

[ref15] Shimoda Y.; Doi Y.; Wakeshima M.; Hinatsu Y. Magnetic and Electrical Properties of Quadruple Perovskites with 12 Layer Structures Ba_4_LnM_3_O_12_ (Ln = rare Earths; M = Ru, Ir): The Role of Metal–Metal Bonding in Perovskite-Related Oxides. J. Solid State Chem. 2010, 183 (9), 1962–1969. 10.1016/j.jssc.2010.06.023.

[ref16] Yang H.; Tang Y. K.; Yao L. D.; Zhang W.; Li Q. A.; Li F. Y.; Jin C. Q.; Yu R. C. Synthesis, Structure and Phase Separation of a New 12R-Type Perovskite-Related Oxide Ba_3_NdMn_2_O_9_. J. Alloys Compd. 2007, 432 (1–2), 283–288. 10.1016/j.jallcom.2006.05.117.

[ref17] Wu J.; Yan X.; Guo W.; Wang X.; Yin C.; Kuang X. Molecule-like Cluster Magnetism and Cationic Order in the New Hexagonal Perovskite Ba_4_Sn_1.1_Mn_2.9_O_12_. RSC Adv. 2021, 11 (63), 40235–40242. 10.1039/D1RA07841K. 35494139 PMC9044838

[ref18] Strange N. A.; Bell R. T.; Park J. E.; Stone K. H.; Coker E. N.; Ginley D. S. Formation of Ba_3_Nb_0.75_Mn_2.25_O_9_-6H during Thermochemical Reduction of Ba_4_NbMn_3_O_12_-12R. Acta Crystallogr., Sect. E: Crystallogr. Commun. 2023, 79 (5), 469–473. 10.1107/S2056989023003213. 37151825 PMC10162086

[ref19] Barcellos D. R.; Sanders M. D.; Tong J.; McDaniel A. H.; O’Hayre R. P. BaCe_0.25_Mn_0.75_O_3−δ_ —a Promising Perovskite-Type Oxide for Solar Thermochemical Hydrogen Production. Energy Environ. Sci. 2018, 11 (11), 3256–3265. 10.1039/C8EE01989D.

[ref20] Fuentes A. F.; Boulahya K.; Amador U. Novel Rare-Earth-Containing Manganites Ba_4_REMn_3_O_12_ (RE = Ce, Pr) with 12R Structure. J. Solid State Chem. 2004, 177 (3), 714–720. 10.1016/j.jssc.2003.08.025.

[ref21] Macías M. A.; Mentré O.; Pirovano C.; Roussel P.; Colis S.; Gauthier G. H. Influence of the Synthesis Route on the Formation of 12R/10H-Polytypes and Their Magnetic Properties within the Ba(Ce,Mn)O_3_ Family. New J. Chem. 2015, 39 (2), 829–835. 10.1039/C4NJ00798K.

[ref22] Macías M. A.; Mentré O.; Colis S.; Cuello G. J.; Gauthier G. H. Structure and Magnetic Properties of Ba_5_Ce_1.25_Mn_3.75_O_15_, a New 10H-Polytype in the Ba–Ce–Mn–O System. J. Solid State Chem. 2013, 198, 186–191. 10.1016/j.jssc.2012.10.004.

[ref23] Trindell J. A.; McDaniel A. H.; Ogitsu T.; Ambrosini A.; Sugar J. D. Probing Electronic and Structural Transformations during Thermal Reduction of the Promising Water Splitting Perovskite BaCe_0.25_Mn_0.75_O_3_. Chem. Mater. 2022, 34 (17), 7712–7720. 10.1021/acs.chemmater.2c00731.

[ref24] Roychoudhury S.; Shulda S.; Goyal A.; Bell R. T.; Sainio S.; Strange N. A.; Park J. E.; Coker E. N.; Lany S.; Ginley D. S.; Prendergast D. Investigating the Electronic Structure of Prospective Water-Splitting Oxide BaCe_0.25_Mn_0.75_O_3−δ_ before and after Thermal Reduction. Chem. Mater. 2023, 35 (5), 1935–1947. 10.1021/acs.chemmater.2c03139.

[ref25] Bell R. T.; Strange N. A.; Plattenberger D. A.; Shulda S.; Park J. E.; Ambrosini A.; Heinselman K. N.; Sugar J. D.; Parilla P. A.; Coker E. N.; McDaniel A.; Ginley D. S. Synthesis and Structure of High-Purity BaCe_0.25_Mn_0.75_O_3_: An Improved Material for Thermochemical Water Splitting. Acta Crystallogr., Sect. B: Struct. Sci., Cryst. Eng. Mater. 2022, 78 (6), 884–892. 10.1107/S2052520622010393.

[ref26] Strange N. A.; Park J. E.; Goyal A.; Bell R. T.; Trindell J. A.; Sugar J. D.; Stone K. H.; Coker E. N.; Lany S.; Shulda S.; Ginley D. S. Formation of 6H-Ba_3_Ce_0.75_Mn_2.25_O_9_ during Thermochemical Reduction of 12R-Ba_4_CeMn_3_O_12_: Identification of a Polytype in the Ba(Ce,Mn)O_3_ Family. Inorg. Chem. 2022, 61 (16), 6128–6137. 10.1021/acs.inorgchem.2c00282. 35404603

[ref27] Streltsov S. V.; Khomskii D. I. Cluster Magnetism of Ba_4_NbMn_3_O_12_: Localized Electrons or Molecular Orbitals?. JETP Lett. 2018, 108 (10), 686–690. 10.1134/S0021364018220071.

[ref28] Tan Z.; Saito T.; Romero F. D.; Patino M. A.; Goto M.; Chen W.-T.; Chuang Y.-C.; Sheu H.-S.; Shimakawa Y. Hexagonal Perovskite Ba_4_Fe_3_NiO_12_ Containing Tetravalent Fe and Ni Ions. Inorg. Chem. 2018, 57 (16), 10410–10415. 10.1021/acs.inorgchem.8b01618. 30067346

[ref29] Ramanathan A.; Kaplan J.; Sergentu D.-C.; Branson J. A.; Ozerov M.; Kolesnikov A. I.; Minasian S. G.; Autschbach J.; Freeland J. W.; Jiang Z.; Mourigal M.; La Pierre H. S. Chemical Design of Electronic and Magnetic Energy Scales of Tetravalent Praseodymium Materials. Nat. Commun. 2023, 14 (1), 3134 10.1038/s41467-023-38431-7. 37253731 PMC10229542

[ref30] Coelho A. A. *TOPAS* and *TOPAS-Academic*: An Optimization Program Integrating Computer Algebra and Crystallographic Objects Written in C++. J. Appl. Crystallogr. 2018, 51 (1), 210–218. 10.1107/S1600576718000183.

[ref31] Zubkov V. G.; Tyutyunnik A. P.; Berger I. F.; Voronin V. I.; Bazuev G. V.; Moore C. A.; Battle P. D. Crystal and Magnetic Structures of Ba_4_Mn_3_O_10_. J. Solid State Chem. 2002, 167 (2), 453–458. 10.1016/S0022-4596(02)99658-1.

[ref32] Rosov N.; Lynn J. W.; Lin Q.; Cao G.; O’Reilly J. W.; Pernambuco-Wise P.; Crow J. E. Antiferromagnetic Ordering of BaPrO_3_ via Neutron Diffraction. Phys. Rev. B 1992, 45 (2), 982–986. 10.1103/PhysRevB.45.982. 10001141

[ref33] Adkin J. J.; Hayward M. A. BaMnO_3-*x*_ Revisited: A Structural and Magnetic Study. Chem. Mater. 2007, 19 (4), 755–762. 10.1021/cm062055r.

[ref34] Adkin J. J.; Hayward M. A. Structure and Magnetism of 4H-BaMnO_3–x_ and 4H-Ba_0.5_Sr_0.5_MnO_3–x_. J. Solid State Chem. 2006, 179 (1), 70–76. 10.1016/j.jssc.2005.09.046.

