# Supplementary material for: Influence of the Rare Earth Cation on the Magnetic Properties of Layered 12R-Ba4M4+Mn3O12 (M = Ce, Pr) Perovskites
Source: Chem Mater. 2024 Mar 9;36(6):2810–8. doi: 10.1021/acs.chemmater.3c03014 (PMC10976642; doi:10.1021/acs.chemmater.3c03014)
Supplement: Supplementary file 1 — cm3c03014_si_001.pdf [file cm3c03014_si_001.pdf]

Supplementary Information for: Influence of the rare earth cation on the magnetic properties of layered 12R-$Ba_4M^{4+}Mn_3O_{12}$ (M = Ce, Pr) perovskites

Michael J. Dzara,[1] Arthur C. Campello,[2,3] Aaron T. Breidenbach,[2,4] Nicholas A. Strange,[5] James Eujin Park,[6] Andrea Ambrosini,[6] Eric N. Coker,[6] David S. Ginley,[1] Young S. Lee,[2,3] Robert T. Bell,[1] Rebecca W. Smaha[1,*]

[1] National Renewable Energy Laboratory, Golden, CO 80401, USA
[2] Stanford Institute for Materials and Energy Sciences, SLAC National Accelerator Laboratory, Menlo Park, CA, 94025, USA
[3] Department of Applied Physics, Stanford University, Stanford, CA, 94305, USA
[4] Department of Physics, Stanford University, Stanford, CA, 94305, USA
[5] Stanford Synchrotron Radiation Lightsource, SLAC National Accelerator, Laboratory, Menlo Park, CA 94025, USA
[6] Sandia National Laboratories, PO Box 5800, Albuquerque, New Mexico 87185, USA

Table of Contents

Structural Information **2**
Additional Magnetism Data **4**
Additional Heat Capacity Data **8**

* Rebecca.smaha@nrel.gov

Structural Information

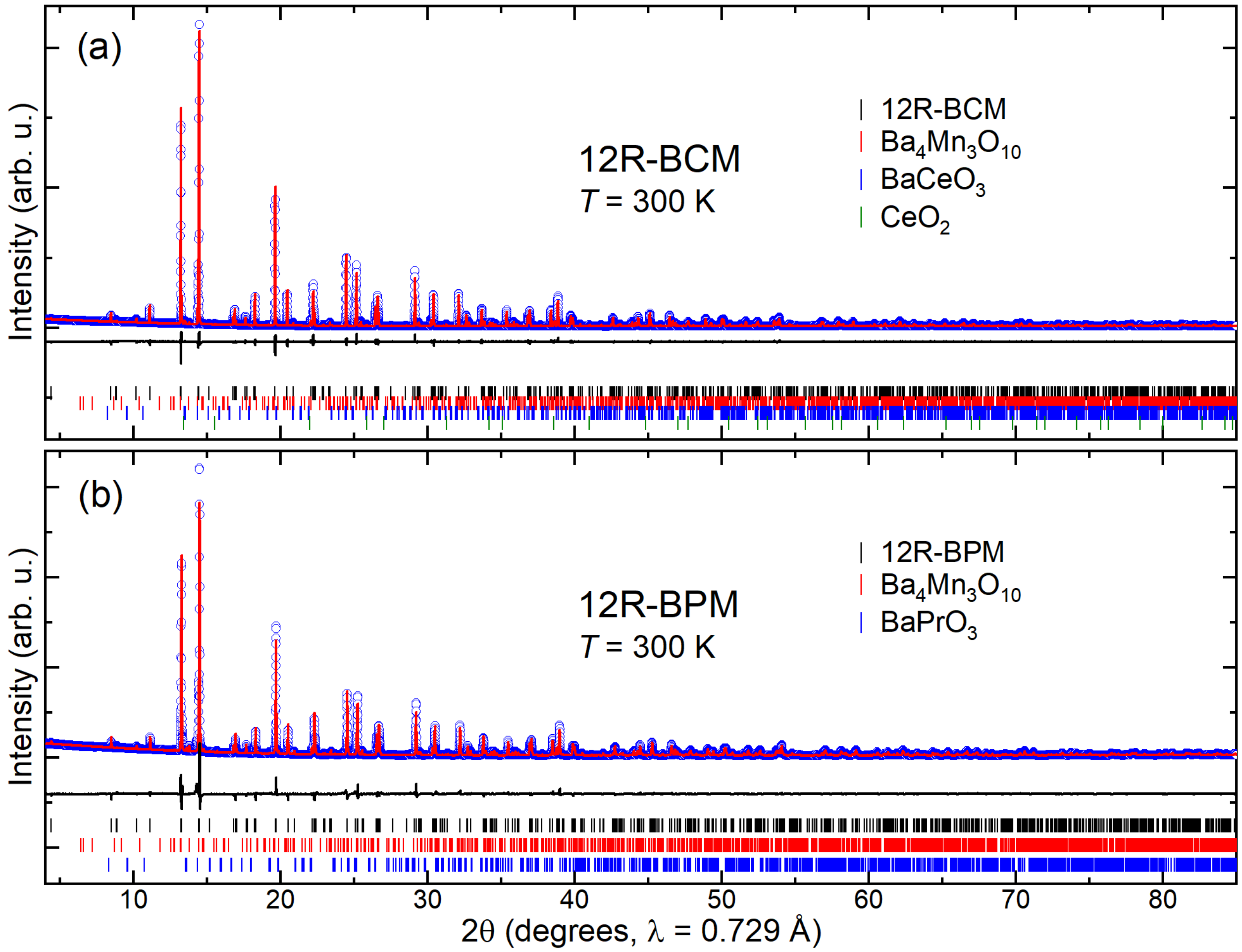

Figure S1: Rietveld refinement of synchrotron PXRD data of (a) 12R-BCM and (b) 12R-BPM at *T* = 300 K. Observed (blue), calculated (red), and difference (black) plots are shown, and Bragg reflections are indicated by tick marks.

Table S1: Crystallographic details elucidated through Rietveld refinements of synchrotron PXRD data in space group *R*-3*m*

**Sample**	**atom**	**Wyckoff site**	**x**	**y**	**z**	**U_{eq} ($Å^2$)**	**Occ.**
**12R-$Ba_4PrMn_3O_{12}$**	Ba1	6c	0	0	0.13117(3)	0.100(19)	1
T = 100 K	Ba2	6c	0	0	0.28274(2)	0.100(19)	1
a = 5.78817(8) Å	Pr1	3a	0	0	0	0.10(2)	1
c = 28.5251(4) Å	Mn1	3b	0	0	0.5	0.10(3)	1
V = 827.64(2) $Å^3$	Mn2	6c	0	0	0.41370(6)	0.10(3)	1
R_{wp} = 7.89%	O1	18h	0.4774(5)	0.5226(5)	0.12576(17)	0.23(5)	1
GOF = 0.40	O2	18h	0.4888(5)	0.5112(5)	0.28911(17)	0.23(5)	1
**12R-$Ba_4PrMn_3O_{12}$**	Ba1	6c	0	0	0.13134(3)	0.38(3)	1
T = 300 K	Ba2	6c	0	0	0.28264(3)	0.38(3)	1
a = 5.79835(10) Å	Pr1	3a	0	0	0	0.10(4)	1
c = 28.5654(5) Å	Mn1	3b	0	0	0.5	0.10(4)	1
V = 831.73(3) $Å^3$	Mn2	6c	0	0	0.41355(7)	0.10(4)	1
R_{wp} = 7.73%	O1	18h	0.4768(5)	0.5232(5)	0.12584(18)	0.45(7)	1
GOF = 0.39	O2	18h	0.4881(6)	0.5232(5)	0.28953(19)	0.45(7)	1
**12R-$Ba_4CeMn_3O_{12}$**	Ba1	6c	0	0	0.13117(2)	0.290(10)	1
T = 100 K	Ba2	6c	0	0	0.28235(2)	0.290(10)	1
a = 5.79249(4) Å	Ce1	3a	0	0	0	0.227(12)	1
c = 28.5881(2) Å	Mn1	3b	0	0	0.5	0.108(14)	1
V = 830.705(13) $Å^3$	Mn2	6c	0	0	0.41355(4)	0.108(14)	1
R_{wp} = 6.21%	O1	18h	0.4785(3)	0.5215(3)	0.12481(10)	0.36(3)	1
GOF = 0.24	O2	18h	0.4892(3)	0.5108(3)	0.28828(10)	0.36(3)	1
**12R-$Ba_4CeMn_3O_{12}$**	Ba1	6c	0	0	0.13126(2)	0.464(14)	1
T = 300 K	Ba2	6c	0	0	0.28233(2)	0.464(14)	1
a = 5.80291(5) Å	Ce1	3a	0	0	0	0.203(15)	1
c = 28.6271(2) Å	Mn1	3b	0	0	0.5	0.142(17)	1
V = 834.834(17) $Å^3$	Mn2	6c	0	0	0.41333(4)	0.142(17)	1
R_{wp} = 5.64%	O1	18h	0.4788(3)	0.5212(3)	0.12479(10)	0.45(3)	1
GOF = 0.27	O2	18h	0.4893(3)	0.5107(3)	0.28832(10)	0.45(3)	1

Table S2: Summary of Phase Fractions (wt. %) extracted from Rietveld refinements of 12R-BCM and 12R-BPM synchrotron PXRD

**Sample**	**Temp.**	**12R-BCM wt.%**	**$Ba_4Mn_3O_{10}$ wt.%**	**$BaCeO_3$ wt.%**	**CeO_2 wt.%**
12R-BCM	100 K	98.395±0.098	0.758±0.079	0.636±0.042	0.211±0.014
12R-BCM	300 K	98.338±0.122	0.790±0.086	0.516±0.045	0.356±0.031
**Sample**		**12R-BPM wt.%**	**$Ba_4Mn_3O_{10}$ wt.%**	**$BaPrO_3$ wt.%**	
12R-BPM	100 K	96.587±0.486	2.472±0.394	0.941±0.161	
12R-BPM	300 K	96.647±0.785	2.387±0.627	0.966±0.264	

Additional Magnetism Data

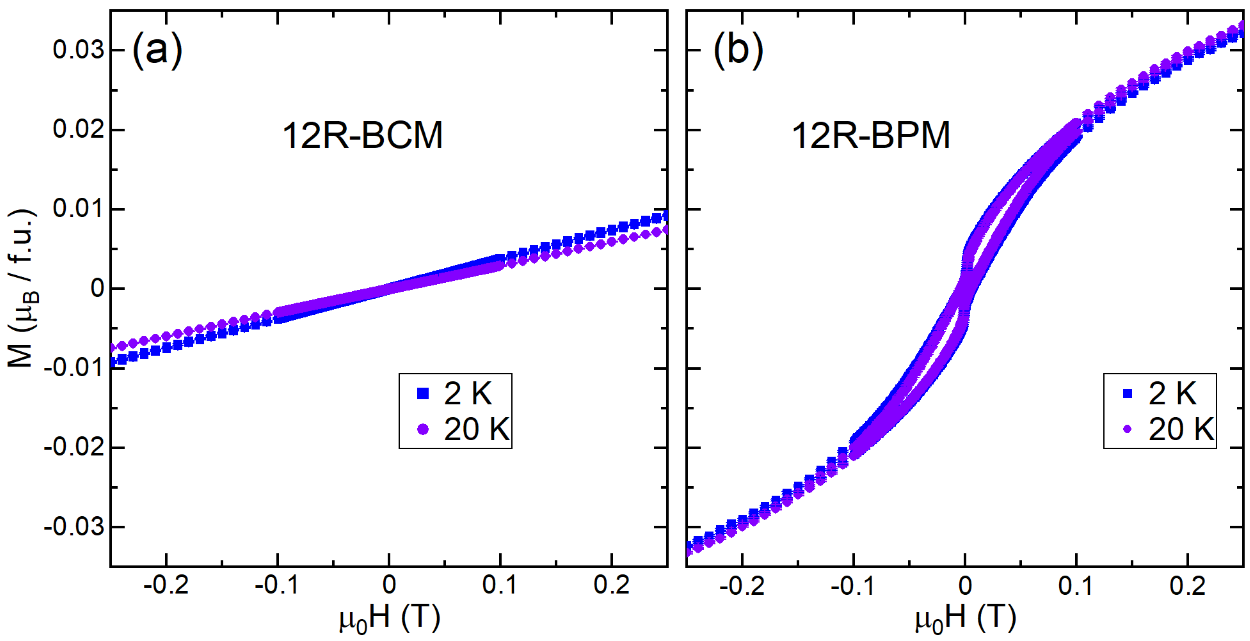

Figure S2: Magnetization (M) as a function of applied field for (a) 12R-BCM and (b) 12R-BPM at $T = 2$ K and $T = 20$ K.

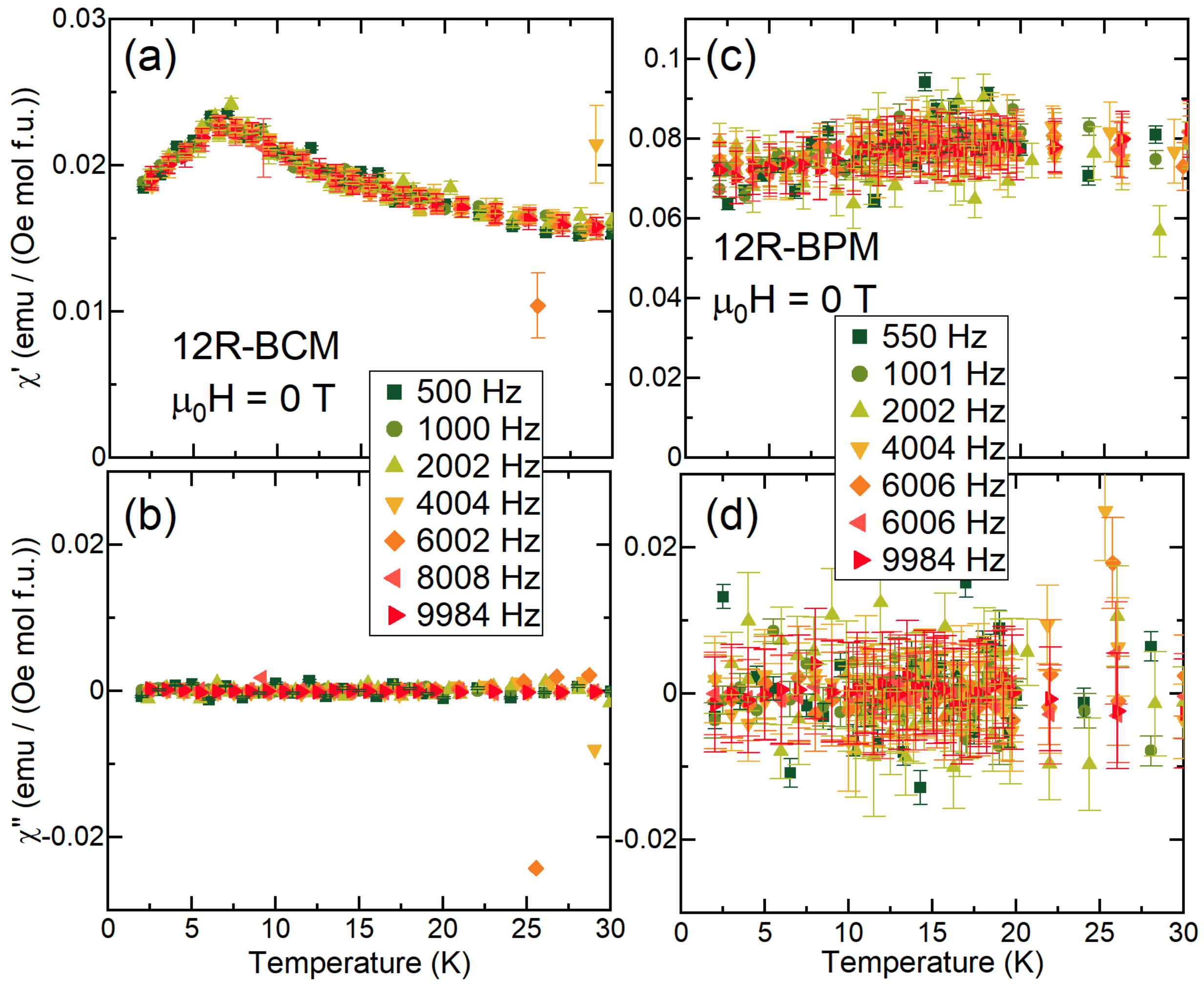

Figure S3: Frequency-dependent AC susceptibility of 12R-BCM and 12R-BPM collected in an applied DC field of $\mu_0 H = 0$ T. (a,b) Real (χ') and imaginary (χ'') parts of the AC susceptibility of 12R-BCM. (c,d) Real (χ') and imaginary (χ'') parts of the AC susceptibility of 12R-BPM.

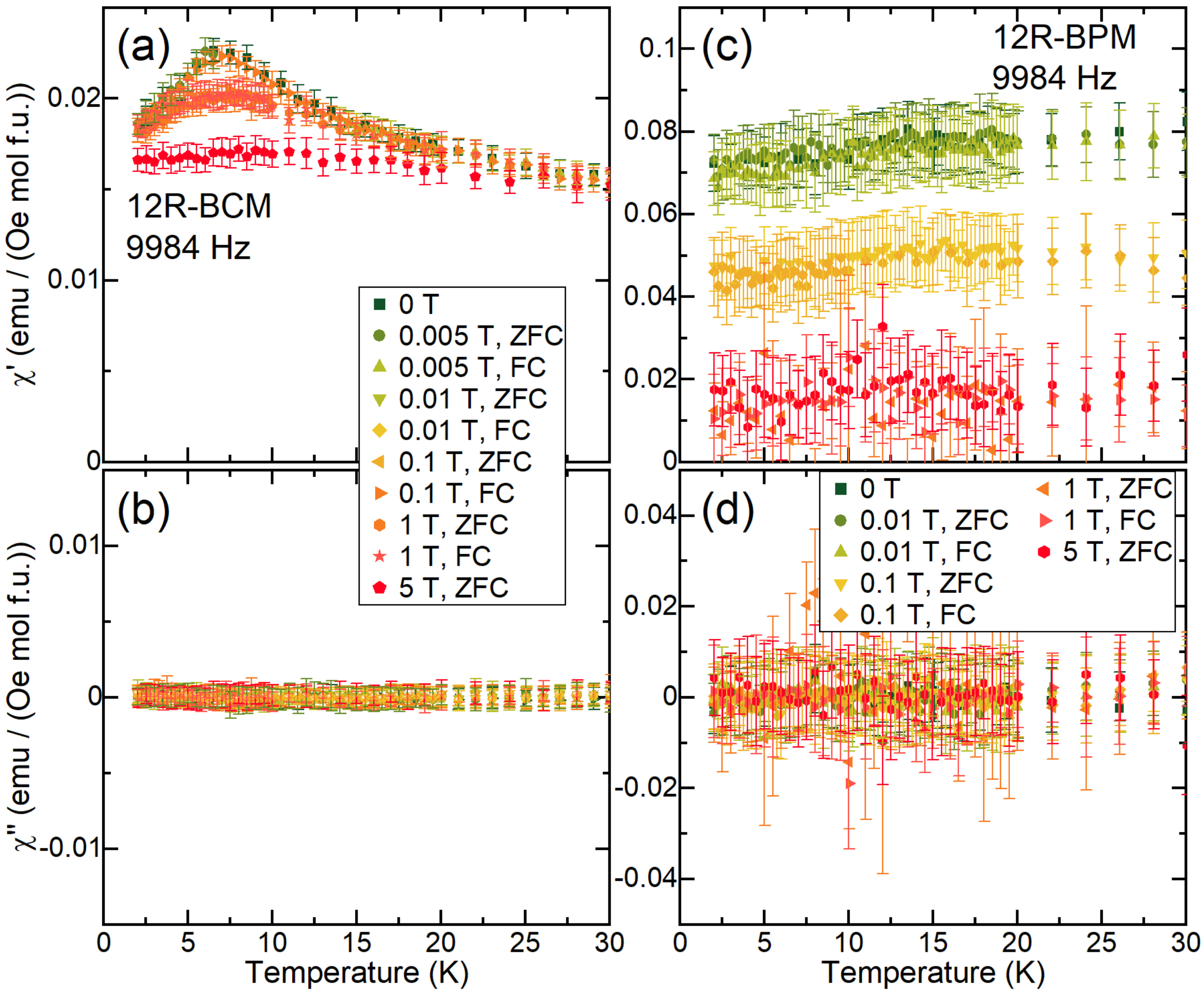

Figure S4: AC susceptibility of 12R-BCM and 12R-BPM collected at a frequency of 9984 Hz in a range of applied DC fields. (a,b) Real (χ') and imaginary (χ'') parts of the AC susceptibility of 12R-BCM. (c,d) Real (χ') and imaginary (χ'') parts of the AC susceptibility of 12R-BPM.

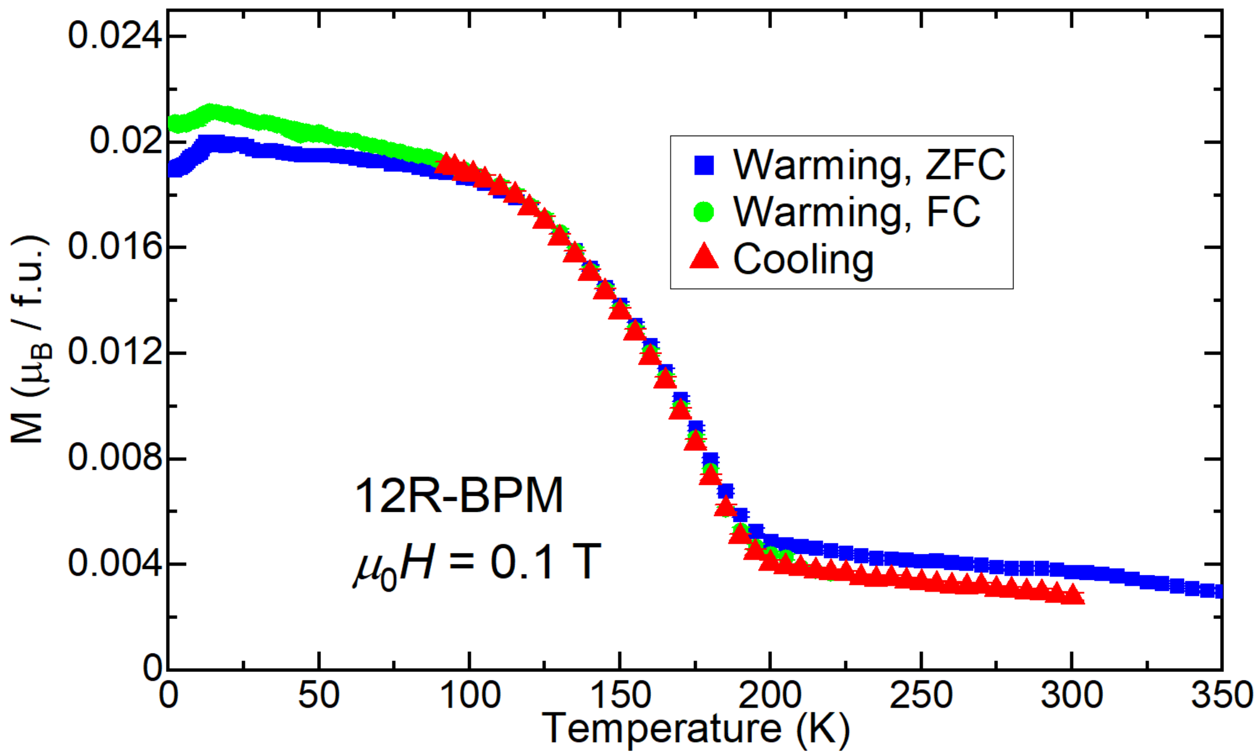

Figure S5: Temperature-dependent magnetization of 12R-BPM collected in an applied field of $\mu_0 H = 0.1$ T upon warming—both zero field cooled (ZFC) and field cooled (FC)—and upon cooling.

Additional Heat Capacity Data

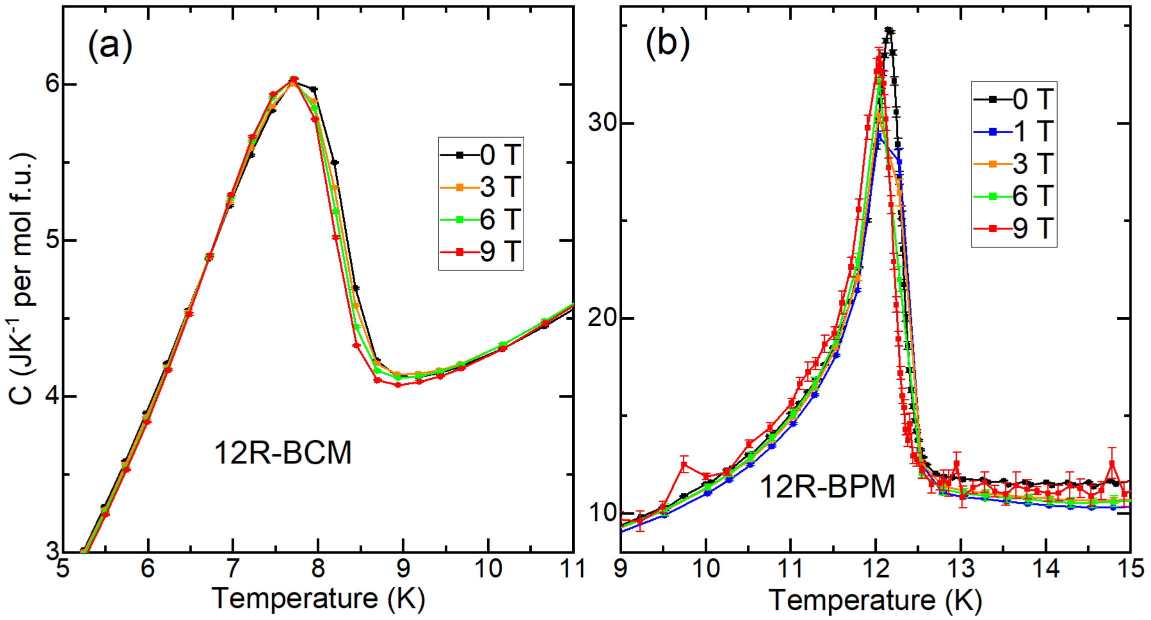

Figure S6: Molar heat capacity (*C*) measurements of A) 12R-BCM and B) 12R-BPM.

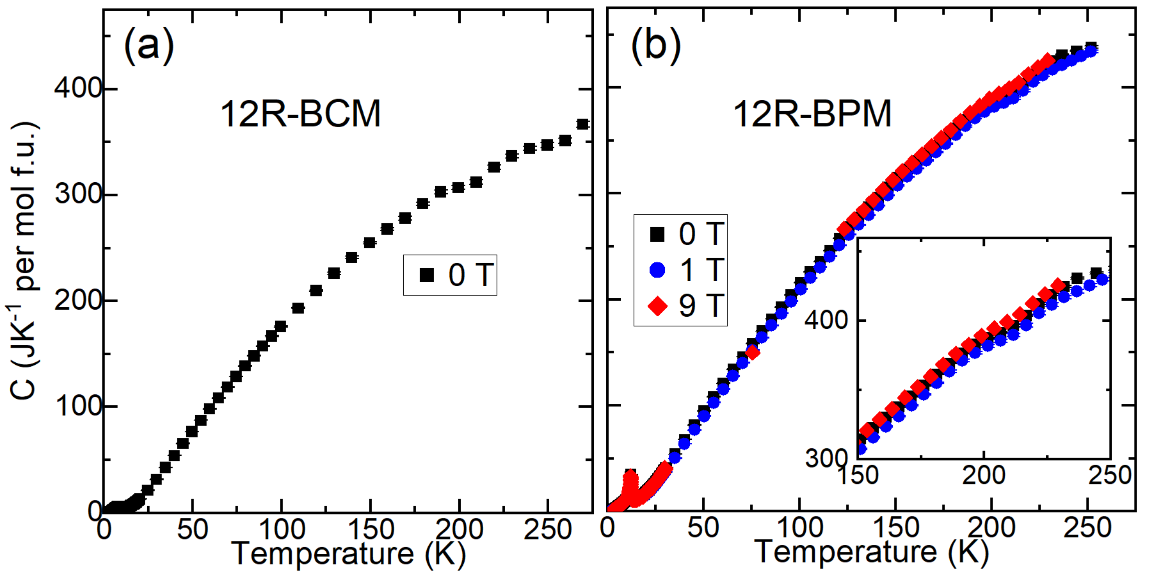

Figure S7: Molar heat capacity (*C*) measurements of (a) 12R-BCM and (b) 12R-BPM up to high temperature.

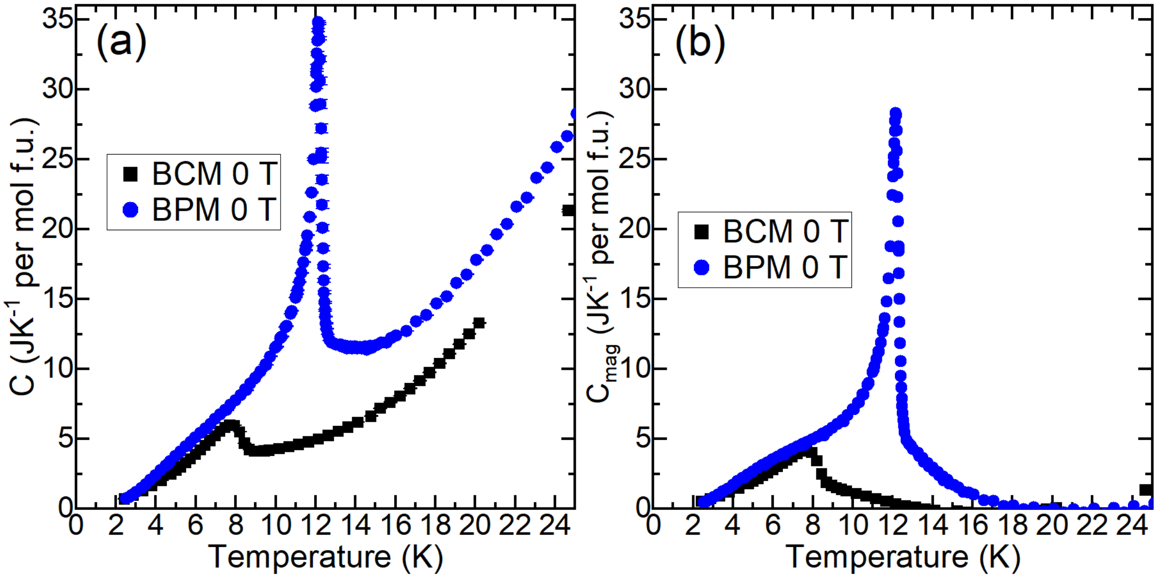

Figure S8: Comparison of (a) molar heat capacity (*C*) and magnetic heat capacity (C_{mag}) of 12R-BCM and 12R-BPM.
